# Effect of Garlic Organic Sulfides on Gene Expression Profiling in HepG2 Cells and Its Biological Function Analysis by Ingenuity Pathway Analysis System and Bio-Plex-Based Assays

**DOI:** 10.1155/2021/7681252

**Published:** 2021-11-30

**Authors:** Chenghao Lv, Caiqiong Wang, Ping Li, Yiwen Huang, Xiangyang Lu, Meng Shi, Chaoxi Zeng, Si Qin

**Affiliations:** ^1^College of Bioscience and Biotechnology, Hunan Agricultural University, Changsha, Hunan 410128, China; ^2^Lab of Food Function and Nutrigenomics, College of Food Science and Technology, Hunan Agricultural University, Changsha 410128, China

## Abstract

Garlic organic sulfides are dietary bioactive components with multiple biofunctions to prevent chronic diseases/inflammation and promote human health. DADS (diallyl disulfide), DATS (diallyl trisulfide), and DTS (diallyl tetrasulfide) are typical organic sulfides with similar structures from garlic. However, the structure-activity relationship of garlic organic sulfides remained unknown. The aim of the present study was to investigate the effect of DADS, DATS, and DTS on the gene expression profiling of human hepatocellular carcinoma cells (HepG2) by application of microarray and specialized analysis software, GO, Bio-Plex-based cytokines assay and IPA and analyze their structure-activity relationship according to antioxidant, anti-inflammatory, and metabolic-related properties. According to the microarray data, with the increase of S atom in garlic organic sulfides, its biological activity was gradually enhanced. In the general catalog of GO, garlic organic sulfides mainly affect biological process, molecular function, and cellular component. RT-qPCR results indicated that the microarray data is trustworthy, and the structure-activity analysis data found that more sulfur atoms have more powerful properties; thus, microarray data of DTS was preceded to the subsequent IPA analysis. The results of IPA analysis showed that the top 5 signaling pathways and molecular functions were disturbed by DTS; the molecular functions with the highest scores affected by DTS are cancer, cell apoptosis, and cell proliferation, which imply that the occurrence or metabolism of these diseases is related to the differential expression of the above-mentioned related genes and the activation of signaling channels, and the core of the most significant molecular network is inflammation. Finally, the results found that the secretions of 6 cytokines in macrophages were significantly inhibited by DTS treatment. This is the first study that analyzed the structure-activity relationship of garlic organic sulfides, which will provide useful genetic information for its multi-biofunction and promote their clinical application in the near future.

## 1. Introduction

Garlic organic sulfides are dietary bioactive components with the functions to prevent chronic diseases/inflammation and promote human health. DADS (diallyl disulfide), DATS (diallyl trisulfide), and DTS (diallyl tetrasulfide) are typical organic sulfides with similar structures to garlic, possessing two, three, and four sulfur atoms, respectively. They are natural compounds with low toxicity, and recent studies found that they have potent bioactive properties such as antioxidation, anticancer, and anti-inflammation activities. However, their structure-activity relationships remained unknown.

Experimental studies have shown that daily consumption of garlic is beneficial to human health, such as reducing the risk of esophageal cancer, gastric cancer, and prostate cancer [1, 2]. A large number of animal studies have shown that garlic has a wide range of biological activities including antioxidant [3, 4], anti-inflammatory, and anticancer activities [5, 6]. In addition, garlic can also be used to treat indigestion and fever, and it has antibacterial property [7]. It is also used as a food preservative in Asia [8].

The main biologically active substance of garlic is garlic organic sulfides (OSCS), which has a pungent smell and spicy taste. Garlic organic sulfur compounds can be divided into fat-soluble organic sulfur compounds and water-soluble organic sulfur compounds. Fat-soluble organic sulfides are classified into monosulfur compounds (DAS), disulfide compounds (DADS), trisulfide compounds (DATS), and tetrasulfide compounds (DTS) according to the number of S atoms [9].

Recent epidemiological investigations have shown that eating garlic can reduce the risk of different types of malignant tumors by inhibiting inflammatory factors [2, 10]. Cell culture experiments have shown that garlic sulfide S-allyl-L-cysteine (SAC) can inhibit the production of NO and the expression of iNOS [11]. In RAW264.7 cells induced by LPS, red garlic organic sulfide can inhibit the production of NO and the expression of iNOS [12]. In BV2 microglia cells stimulated by LPS, DADS can also inhibit the production of NO and PGE2 [13]. In addition, Liu et al. compared the effects of DAS, DADS, and DATS on the inhibition of NO production and iNOS expression and found the inhibitory effects in the order of DATS>DADS>DAS, which indicates that the inhibitory effect may be similar to that of S in garlic organic sulfide [14]. In RAW264.2 cells stimulated by LPS, DATS exhibited the best inhibitory effect on inflammation when compared to GSAC, SAC, DAS, and DADS [15]. Therefore, the structural difference of organic sulfide in garlic may be the key factor affecting its biological activity.

Garlic organic sulfides also exert anti-inflammatory effects by inhibiting the expression of proinflammatory factors. Allicin can inhibit the expression of proinflammatory factors induced by TNF-ɑ [16]. DADS can also inhibit the expression of proinflammatory factors, induced by LPS, such as IL-1*β* and TNF-*β* [13]. In RAW264.7 cells induced by LPS, DATS inhibited the expression of IL-6, IL-10, IL-12, KC, MCP-1, and TNF-ɑ [15].

Experimental data from several studies indicate that the expression of proinflammatory factors such as iNOS and COX2 is related to the NF-*κ*B signaling pathway. DADS was reported to attenuate the expression of the NF-*κ*B signaling pathway [17]. However, DADS can inhibit the expression of transcription factors p65, c-Jun, and c-fos in the nucleus [18]. On the other hand, allicin can downregulate the transcription factors p65 of the upstream kinase IKK*α*/*β* and downregulate I*κ*B and attenuate the nuclear translocation of p65 [15]. These experimental studies show that DATS can inhibit the inflammatory response induced by LPS and downregulate the IKK-mediated NF-*κ*B signaling pathway.

Mitogen-activated protein kinases (MAPKs), including JNK, p38, and ERK, play a key role in the inflammatory responses [19]. Therefore, in some chronic inflammatory diseases including rheumatoid arthritis, psoriasis, inflammatory bowel disease, and chronic obstructive pulmonary disease, inhibiting the expression of MAPKs is an effective treatment. Some studies have shown that DADS can attenuate the MAPK signaling pathway induced by LPS and thereby slowing down the inflammatory responses [13]. Allicin can inhibit the ERK signaling pathway and thereby inhibit the proliferation of cancer cells [20]. DADS and DATS can inhibit the activity of AP-1 mediated by JNK and ERK and promote the expression of GST protein and mRNA [21]. DATS attenuates AP-1 activity and COX-2 expression by inhibiting JNK and Akt signaling pathways [22]. DATS can also inhibit the phosphorylation of TAK1, the common upstream regulator of IKKɑ/*β* and MAPK signaling pathways [13, 15]. In addition, it also inhibits the phosphorylation of AKT1, an upstream protein kinase that activates IKK [23, 24]. These experiments show that garlic organic sulfide can inhibit the MAPK and NF-*κ*B signaling pathway mediated by AKT1/TAK1 and thereby inhibiting the inflammatory responses.

In this study, we investigated the effects of DADS, DATS, and DTS on the gene expression profiling of human hepatocellular carcinoma cells (HepG2) by application of microarray and specialized analysis software, GO and IPA, and analyzed their structure-activity relationship according to antioxidant, anti-inflammatory, and metabolic-related properties. Finally, LPS-induced RAW264.7 macrophages were treated with DTS to verify its inhibitory effect on inflammation and maintenance of redox balance.

## 2. Materials and Methods

### 2.1. Cell and Cell Cultures

HepG2 cells were cultured in DMEM medium containing 10% fetal bovine serum (FBS), and the temperature was maintained at 37 cu with stable 5% CO_2_ gas supply. Discard the old culture medium in the 25 cm^2^ petri dish, add about 1 ml 0.01 M PBS with a pipette, shake gently to rinse the cell surface, and wash away the remaining FBS to prevent it from affecting the trypsin digestion. Use the pipette tip to suck off the PBS (not directly poured), add 1 ml of 0.05% pancreatin solution to cover the bottom of the bottle, incubate for 1 minute, then discard most of the pancreatin, and keep it at 37 t in an incubator for 2 minutes. By using the inverted microscope, the digestion of the cells was observed. It was observed that the cells gradually shrink and became rounded, the cell connection disappeared, the spacing increased, and a few cells were fallen off the bottle wall. At this time, we quickly added 2 ml fully grown DMEM/F12 medium (containing 10% FBS), gently pipetted with a disposable sterile straw to make it into a cell suspension. Transfer the digested cells into a centrifuge tube, and centrifuge it at 1,000 rpm or 800 rpm for 5 minutes. Aspirate the supernatant with a pipette, to the cell pellet adding 3-4 ml DMEM/F12 medium containing 10% FBS to fully suspend the cells (gently mix the front, back, left, and right); if necessary, add antibiotics (commonly used penicillin), and then put them into 3-4 petri dishes to make the total amount of liquid in each bottle reach 5 ml. Keep the well-mixed cell culture dish carefully into an incubator at 37 with 5% CO_2_, and continue culturing.

### 2.2. Cell Toxicity Determination

MTT assay was used to check the cytotoxicity of DSs. HepG2 cells were seeded on a 96-well plate at a density of 104 cells per well and then preincubated at 37°C for 24 h; then the specified dose (0.5 *μ*M, 10 *μ*M, and 20 *μ*M) of garlic organic sulfide compound was added and incubated for another 24 h. Then, add MTT to the plate (final concentration is 0.5 mg/ml) and incubate for 4 h, add acidic isopropanol (HCl in isopropanol is 0.04-0.10 N) to dissolve formazan crystals, and use a microplate reader (Thermo Scientific, USA) to measure the optical density (OD) at 575 nm. Cell viability was determined by comparing the OD value of DSs-treated cells with that of untreated cells.

### 2.3. RNA Extraction and Microarray

HepG2 cells were precultured in a petri dish for 24 hours and then treated with DADS, DATS, and DTS at a concentration of 10 *μ*M dissolved in 0.1% DMSO for 10 hours. Total RNA was extracted using the Isogen RNA kit (Nippon Gene Co., Tokyo, Japan) according to the instructions, and the RNA quality was evaluated by automatic capillary gel electrophoresis on the Agilent Bioanalyzer 2100 (Palo Alto, CA, USA). These total RNA samples were labeled according to the standard single-cycle amplification, and labeling protocol was developed by Affymetrix (Santa Clara, CA, USA), and cRNA was labeled at 40°C for 2 hours. After amplification and labeling, the Affymetrix Gene Chip Human U133 plus 2.0 Array system was used for gene chip testing. The Affymetrix Gene Chip Human U133 plus 2.0 chip contained more than 44 K oligonucleotides, and the hybridization fluorescence was scanned using the Affymetrix scanner.

### 2.4. GO Ontology and IPA Analysis of Microarray Data

The gene chip results were first classified according to the Gene Ontology ID (GO ID) (http://www.geneontology.org/). Gene Ontology is a major bioinformatics method that is aimed at standardizing genes and gene products across species and database representation of attributes. Then, through the Ingenuity Pathway Analysis (IPA) system (http://www.ingenuity.com) for analysis.

### 2.5. Bio-Plex-Based Assays of Inflammatory Cytokine

HepG2 cells were precultured and starved accordingly to eliminate the effect of FBS. The cells were treated with 10-20 *μ*M of DTS for half hour before exposure to 1 *μ*g/ml LPS for additional 12 h. The 27 cytokines' detection, including IL-1*α*, IL-1*β*, IL-2, IL-4, IL-5, IL-6, IL-7, IL-8, IL-9, IL-10, IL-12, IL-13, IL-15, IL-17, RANTES, PDGF-BB, FGF basic, IP-10, TNF-*α*, Eotaxin, IFN-*γ*, MCP-1, MIP-1*α*, MIP-1*β*, GM-CSF, and VEGF, was performed by using Bio-Plex Pro Human Cytokine 27-Plex Panel kit (Bio-Rad Laboratories) and Bio-Plex cytokines assay system (Bio-Plex 200, Bio-Rad) according to the manufacturer's instructions, and the results were analyzed by the Bio-Plex manager software (version 4.0).

### 2.6. Statistical Analysis

All the experimental data in the present study were repeated at least three or four times. Significances or differences of treated vs. control were analyzed by using Student's *t*-test, and *p* < 0.05 was considered significant.

## 3. Results and Discussions

### 3.1. Gene Expression Profiling

In the source data of the gene chip by DADS treatment, the expression of 3 genes changed by 5 times or more, and the expression of all 3 genes was upregulated. The fold change of the expression of 2 genes was between 4 and 5 times, and the expression of all 2 genes was upregulated. The expression changes of 13 genes were between 3 and 4 times, and the expression of all 13 genes was upregulated. The expression of 110 genes varied from 2-fold to 3-fold, 85 of which were upregulated and 25 were downregulated. In the source data of the experimental group treated by DADS, out of 54675 genes, there were 128 genes (accounting for 0.23%, 128 : 54675) that showed the expression change higher than 2 times (Table 1).

In the source data of the gene chip by DATS treatment, the expression of 4 genes had a fold change greater than or equal to 5 times, the expression of 3 genes was upregulated, and the expression of 1 gene was downregulated. The expression changes of 7 genes were between 4-fold and 5-fold, 6 genes were upregulated, and 1 gene was downregulated. The expression changes of 32 genes were between 3 times and 4 times, 18 genes were upregulated, and 14 genes were downregulated. The expression of 201 genes varied between 2-fold and 3-fold, 99 of which were upregulated and 102 genes were downregulated. In general, in the source data of the experimental group processed by DATS, 244 genes (0.45%, 244 : 54675) out of 54675 genes had expression changes higher than 2 times (Table 1).

In the source data of the gene chip by DTS treatment, the expression of 4 genes changed by 5 times or more, and the expression of all 4 genes was upregulated. The expression changes of 8 genes were between 4-fold and 5-fold, 7 genes were upregulated, and 1 gene was downregulated. The expression changes of 29 genes were between 3 times and 4 times, 21 genes were upregulated, and 8 genes were downregulated. The expression of 203 genes varied between 2-fold and 3-fold, of which 115 genes were upregulated and 88 genes were downregulated. In general, in the source data of the experimental group processed by DTS, there were 244 genes (0.45%, 244 : 54675) out of the 54675 genes whose expression has changed more than 2 times (Table 1).

The number of downregulated genes in the DATS group was more than that in the DTS group. However, the number of genes upregulated in the DTS group is significantly higher than that in the DATS group and gene expression of the DADS group changed more than 2 times, and the number of upregulated genes increased in the order of DTS>DATS> DADS.

### 3.2. GO Ontology Analysis of Microarray Data

In this study, we first used the free analysis tool GO (Gene Ontology) on the whole network to analyze the gene chip data. This analysis method is based on one or more GO numbers corresponding to each gene to find each gene in the official catalog of GO categories, and it has the following three major categories, namely, biological process, cellular component, and molecular function. Each major category contains 6 subcategories. For classification, each category also has a corresponding GO number. Since this experiment was intended to study the results of the differential expression of hepatocarcinoma genes after treatment with DADS, DATS, and DTS and their relationship with physiological activity, only genes with significant changes in differential expression are selected for the overall analysis. According to the results of previous studies, we selected genes whose changes were greater than 2 times for statistical analysis. There were 616 genes whose expression changes were greater than 2 times, accounting for 1.13% of the total number of genes. The results of GO analysis showed that there are 32, 27, and 25 subgroup genes are disturbed by garlic organic sulfides treatment in the total three categories including biological processes, cell composition, and molecular function. The most significantly disturbed genes are related to protein binding in DTS group, which indicated that DTS can significantly affect the intracellular signal pathway.

### 3.3. DADS/DATS/DTS Regulates the Expressions of Genes

#### 3.3.1. DADS/DATS/DTS Regulates the Expressions of Liver Drug Metabolizing Enzymes

To understand the effect of garlic organic sulfide on HepG2 cells and predict its metabolism in the liver, we studied the gene expression changes of drug metabolizing enzymes and transporters in cells treated with DADS, DATS, and DTS. Phase I enzymes are mainly responsible for binding heterologous substances to carriers on the cell surface during drug metabolism. Phase II enzymes function to enhance their water solubility or expose specific binding sites to facilitate binding to transporters. Transport enzymes are to transport the foreign material out of the cell and excrete it from the body through the blood circulation. In this study, changes were observed in gene expression of 35 drug metabolizing enzymes and transporters. Coenzyme I related to typical phase I enzymes represents FMO5 and NCF2, and related to phase II coenzymes, it is worth noting that the differentially expressed phase II coenzymes also include GCLC, GCLM, SQSTM1, and other antioxidant enzymes. There are 10 genes related to proteins such as the ATP and SLC families (Table 2). In the DTS treatment group, the upregulated expression of phase I coenzyme was more than 2 times that of NCF2. GCLC, GCLM, SQSTM1, and HMOX1 were found to be more than 2 times upregulated in phase II coenzyme or antioxidant protein. SlC6A6 and SLC2A2 are the genes that express more than 2 times in transporters.

#### 3.3.2. DADS/DATS/DTS Regulates the Expressions of Inflammation-Related Genes

To understand the effect of garlic organic sulfide on HepG2 cells and predict its effect on inflammation in the liver, we studied the expression changes of inflammation-related genes in cells treated with DADS, DATS, and DTS. The typical inflammatory cell pathways affected by DSs are the NF-*κ*B signaling pathway, IL-6 signaling pathway, and IL-2 signaling pathway. According to gene chip data, gene expression changes more than 2 times, and genes related to inflammatory cell pathways include TLR6, FOS, SOCS1, BCL10, and TNFAIP3. Among them, DSs had the greatest impact on the expression changes of FOS genes. The expression changes of FOS genes in the DTS treatment group were higher than 4 times, and both the DADS and DATS experimental groups were more than 3 times (Table 3).

#### 3.3.3. DADS/DATS/DTS Regulates the Expressions of Genes Related to Glucose and Lipid Metabolism

To understand the effect of garlic organic sulfides on glycolipid metabolism, we studied changes in the expression of genes related to glycolipid metabolism in cells treated with DADS, DATS, and DTS. Among the genes related to glucose and lipid metabolism, GCLC, GCLM, SLC2A2, and GCKR showed more than 2 times of expression changes after DS_S_ treatment. In the DATS and DTS groups, the expression of SLC2A2 gene changed more than 3 times and the gene was downregulated (Table 4).

#### 3.3.4. DADS/DATS/DTS Regulates the Expression of Genes Related to Cell Proliferation and Apoptosis

To understand the effect of garlic organic sulfide on cell proliferation and apoptosis, we investigated the changes in gene expressions related to cell proliferation and apoptosis in HepG2 cells treated with DADS, DATS, and DTS. After DSs treatment, the expression of apoptosis-related genes changed more than 2 times, including F2RL2, HSPA1A///HSPA1B, GCLC, GCLM, SLC2A2, EGR1, FOS, BCL10, TLR6 and NCF2. According to the data from the gene chip, DSs can promote the expression of apoptosis genes, especially the F2RL2 gene, HSPA1A///HSPA1B gene, FOS gene, and NCF2 gene. The gene expression change multiples are more than 3 times, and the F2RL2 gene expression within the multiple is even more than 14 times. DSs can also downregulate the expression of SLC2A2 gene. The expression of cell proliferation-related genes changed more than 2-fold, including F2RL1, SOCS1, FOS, FOSL1, HSPA1A, JAG1, and SPP1 genes. From the data of the gene chip, we can see that the most typical gene that affects cell proliferation is the F2RL2 gene (Table 5).

#### 3.3.5. Real Time Quantitative-PCR Analysis

To verify the accuracy of the gene chip results and compare the structure-activity relationship of the three compounds, we further verified the expression of typical drug metabolizing enzymes and antioxidant protein-related genes in HepG2 cells treated with the above three organic sulfides of garlic and analyzed whether the number of sulfur atoms is the same as the change trend of the expression of the genes.

**Figure 1:**
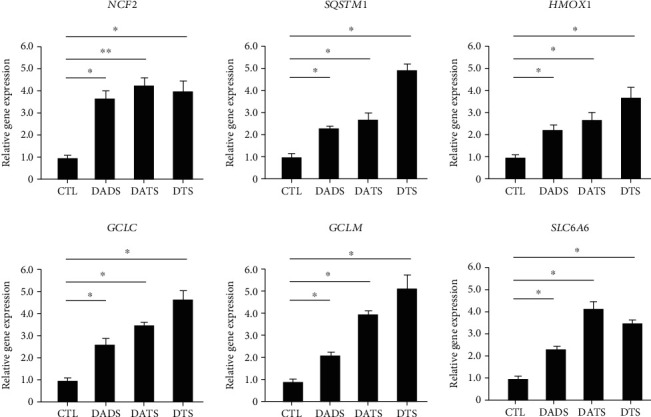
RT-PCR results of typical genes in HepG2 cells treated with DADS/DATS/DTS.

After treating HepG2 cells with the same concentrations of the samples, the total RNA was extracted and then reverse transcribed into cDNA. Finally, the target genes were detected with a fluorescent kit and a real-time fluorescent quantitative PCR. Statistical analysis of the data can provide useful information. Due to the high cost of RT-PCR experiments, only 6 typical genes for drug metabolizing enzymes and antioxidant proteins were selected for RNA transcription level verification. The selected genes were NCF2, SQSTM1, HMOX1, GCLC, and GCLM. And SLC6A6 belongs to the genes corresponding to phase I/II antioxidant proteins and phase III transporter (Figure 1).

The selected genes showed similar expressions between the DNA chip and RT-PCR data, and the trend of differential expression of the genes was the same. Among them, the expression level of anti-inflammatory factor IL11 increased by 4.21 times in real-time PCR, while it increased by 3.33 times in the DNA chip. The reason for the difference in expression multiples is that the results of RT-PCR were obtained from real-time fluorescence measurement, which can record more minutes and precise gene differential expression in real time, so the measured difference multiple is larger and more precise. Compared with treatment by DADS and DATS, DTS can significantly upregulate the expression of SQSTM1, HMOX1, GCLC, and GCLM. These data indicate that treatment of HepG2 cells by DTS can indeed enhance the expression of their antioxidant genes, reduce inflammation, and increase the expression of genes related to metabolic regulation.

### 3.4. IPA Analysis of Microarray Data

To further analyze the potential relationship between significantly changed genes and biological functions, we input the gene chip data into the IPA system, and then the system outputs an analysis report: including typical pathways that are significantly affected, molecular networks, biological functions, and molecules; due to the limitation of study, only some important issues were selected for analysis in this part.

#### 3.4.1. Main Typical Affected Signaling Pathways

The typical approach affected by DTS processing can help locate its main signal channel. The most affected signal channel in the DTS treatment group is glutamate metabolism, and its *p* value takes the negative logarithm to the maximum value of 3.47; the second affected channel is acute phase response signal.

**Figure 2:**
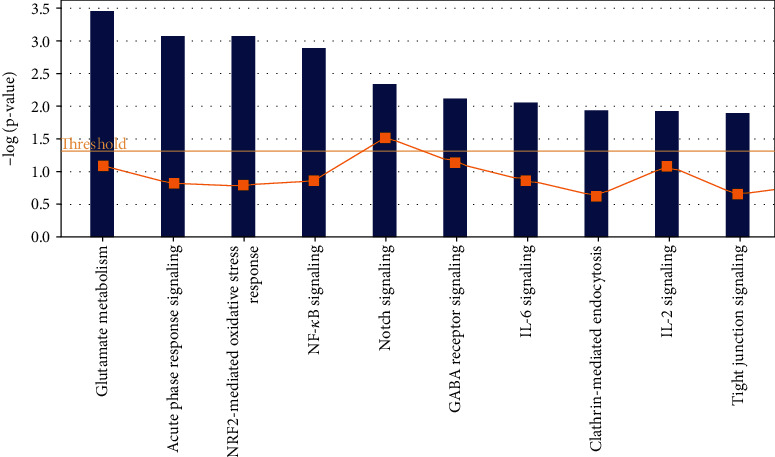
Top 10 typical cell pathways affected by DTS treatment.

The *p* value of NRF2-mediated oxidative stress response activated by DTS takes a negative logarithm of 3.08. It is a very essential and complex signal transmission pathway. Nuclear factor Nrf2 (also called Nfe212) is a transcription factor that regulates the redox state of cells. Nrf2 has the ability to promote the expression of various protective genes of cells in response to various stimuli (including redox signals, inflammation, growth factors, and energy supply changes), which makes it play a key role in cell adaptation. Experimental evidence shows that Nrf2 can profoundly affect metabolism: Nrf2 protects the liver from steatosis by promoting fatty acid oxidation and inhibiting adipogenesis and can promote the formation of fat in peripheral tissues by activating C/EBPb and PPARg. The effect of Nrf2 on lipid metabolism and its obvious ability to inhibit gluconeogenesis indicate that CNC-bZIP factor contributes to metabolic flexibility; that is, it helps to promote organism's lipid oxidation and carbohydrate oxidation as an energy ability to convert. Therefore, the effects of DTS on the metabolism of foreign substances, C21-steroid hormones, glycerophospholipids, glycerolipids, and glutamate may all be related to NRF2, which further confirms that the expression of drug metabolism enzymes is significantly affected by DTS treatment. At the same time, Nrf2 can also be used as a stress-activated transcription factor that mediates drug metabolism enzymes. Nrf2 can control the expression of many phase I and phase II drug metabolism enzymes, as well as other multidrug resistance-related protein (MRP) transporters including MRP2, MRP3, MRP4, and MRP5 expressions. In addition to regulating the basic and induced expression of drug metabolism enzymes, Nrf2 also controls the key components of the endogenous antioxidant system: many drug metabolism enzymes and antioxidant systems require NADPH as a cofactor, including aldehyde ketone reductase (AKR), NQO1, GSR1, and TXNRD1, and it is worth noting that NADPH produces enzymes glucose-6-phosphate dehydrogenase (G6PD), 6-phosphogluconate dehydrogenase (PGD), isocitrate dehydrogenase (IDH)1, and malic enzyme (the processes of ME)1 which are regulated by Nrf2. Therefore, the high activity of Nrf2 can ensure that the expression of the enzyme-catalyzed reduction reaction is combined with the supply of cofactors. In addition, other signal channels that can be significantly activated in the DTS treatment group are NF-*κ*B signaling, IL-6 signaling, and IL-2 signaling. These three cell pathways are all inflammatory cell pathways. Therefore, DTS can also activate inflammatory cell pathways, showing its anti-inflammatory potential (Figure 2).

#### 3.4.2. Top 10 Molecular Functions Changed by DTS Treatment

**Figure 3:**
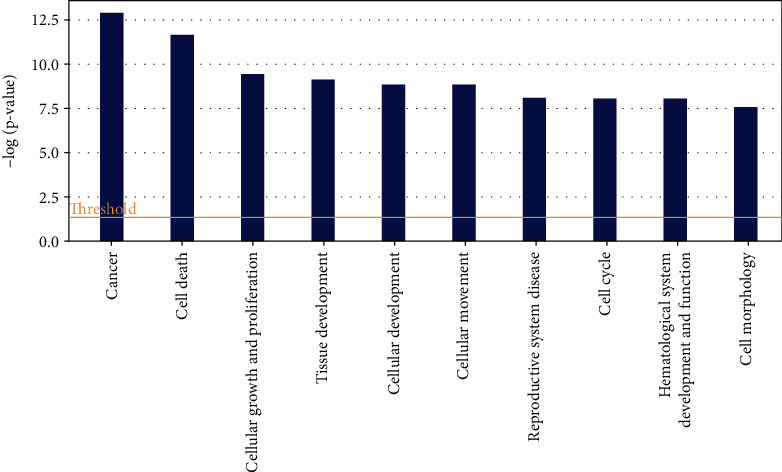
Top 10 molecular functions changed by DTS treatment.

The 10 major molecular functions affected by DTS are Cancer, Cell Death, Cellular Growth and Proliferation, Tissue Development, Cellular Development, Cellular Movement (Cell Movement), Reproductive System Disease, Cell Cycle, Hematological System Development and Function, and Cell Morphology. DTS changed the expression of F2RL1, SLC2A2, FOS, PALLD, TRIB1, and other genes more than 2 times. DTS can significantly affect cell proliferation and apoptosis and cell cycle arrest. In addition, the expression changes of DTS on cancer-related genes such as FOSL1, JAG1, FLRT2, and BCL10 are also more than 2-fold, indicating that DTS may have an anticancer effect (Figure 3).

#### 3.4.3. Molecular Network and Signal Channel Analysis

The 10 most significant molecular networks after DTS treatment are shown in Table 3. Among them, the most affected by DTS are Gene Expression, Protein Synthesis, and Cell Death (scoring: 49 points). Three main biological functions are involved in this network: gene expression, protein synthesis, and cell apoptosis, followed by Cardiovascular System Development and Function, Tissue Morphology, and Amino Acid Metabolism (scoring: 39 points).

To determine the relevant molecular networks and signal channel of differentially expressed genes, in this study, we used the IPA biological software to perform typical signal channels and network analysis on the upregulated, downregulated, and integrated data sets and constructed a comparison between the DTS treatment group and the non-DTS treatment control group. The captions illustrate the classification attributes of molecules of different shapes. The metabolites marked in red in the figure are upregulated metabolites, and the metabolites marked in green are downregulated metabolites. The solid line represents the direct interaction between the two molecules, and the dashed line represents the indirect interaction between the two molecules. Relationship, networks, and signal channels describe the functional relationships between gene products based on known interactions reported in the literature.

There are 35 genes involved in the construction of the most significant molecular network affected by DTS; 25 genes are differentially expressed. NF-*κ*B, as the central position gene of this molecular network, controls the transcriptional activity of multiple inflammatory factors and the expression of antioxidant genes. Among them, there are 12 upregulated genes, and the most significant gene of upregulated fold change is HSPA1A. There are 13 downregulated genes, and the gene with the most significant downregulation fold change is BIRC3. In the molecular network, the upregulated expression of HSPA1A was indirectly affected by NF-*κ*B, which had the largest fold change of 3.8. It promotes the correct folding of newly translated and misfolded proteins and stabilizes or degrades mutant proteins. It helps DNA repair. Its functions contribute to biological processes, including signal transduction, apoptosis, protein homeostasis, cell growth, and differentiation. It is associated with a large number of cancers, neurodegenerative diseases, cellular senescence and aging, and inflammatory diseases such as diabetes type 2 and rheumatoid arthritis.

**Figure 4:**
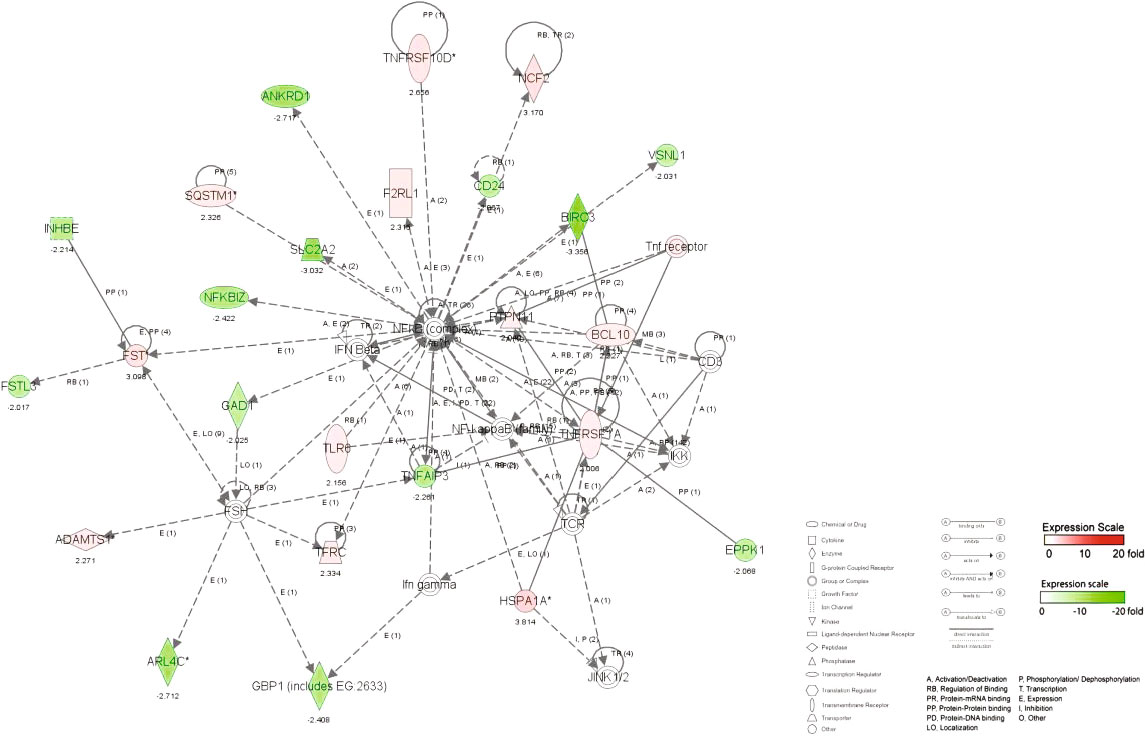
The most significant molecular network affected by DTS.

In this molecular network, NCF2 (neutrophil cytoplasmic factor 2) is upregulated after being indirectly affected by NF-*κ*B. NCF2 is also called NADPH oxidase and is a multiprotein complex with 67 kilodalton molecular subunits. It is related to chronic diseases, especially chronic inflammation. The deletion induced by Alu duplication in the NCF2 gene is a new mechanism that causes p67-hypoxic chronic granulomatous disease (CGD). Chronic granulomatosis is associated with life-threatening infections and disorders. The downregulated expression of BIRC3 was indirectly affected by NF-*κ*B, and the fold change was the largest, which was 3.35. BIRC3 is a member of the apoptosis family, which inhibits cell apoptosis by interfering with the activation of caspase (Figure 4).

**Figure 5:**
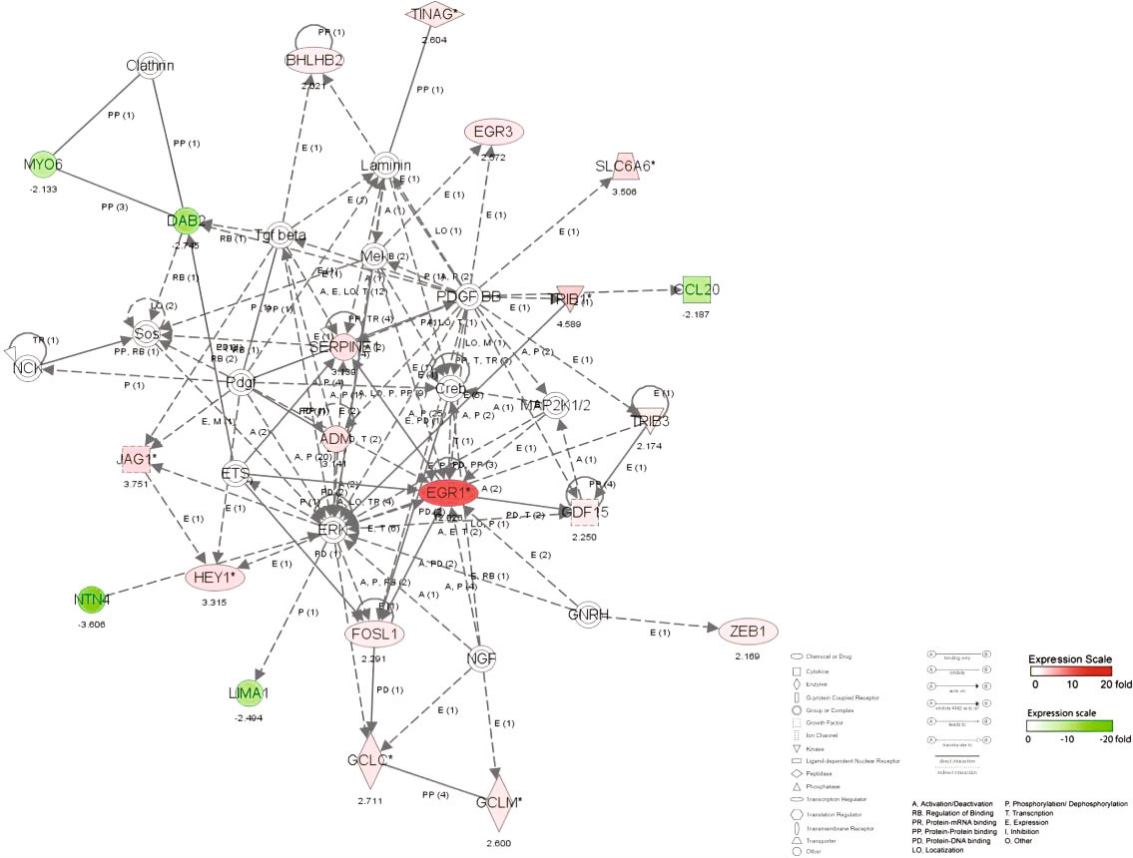
The second most significant molecular network affected by DTS.

There are 35 genes involved in the construction of the second most significant molecular network, of which 21 genes are differentially expressed. ERK, as the central position gene of this molecular network, controls the transcription activity of multiple inflammatory factors and the expression of antioxidant genes. Among them, there are 16 upregulated genes, and the gene with the most significant upregulated fold change is EGR1. There are 5 downregulated genes, and the most significant gene of downregulated fold change is NTN4 (Figure 5).

**Figure 6:**
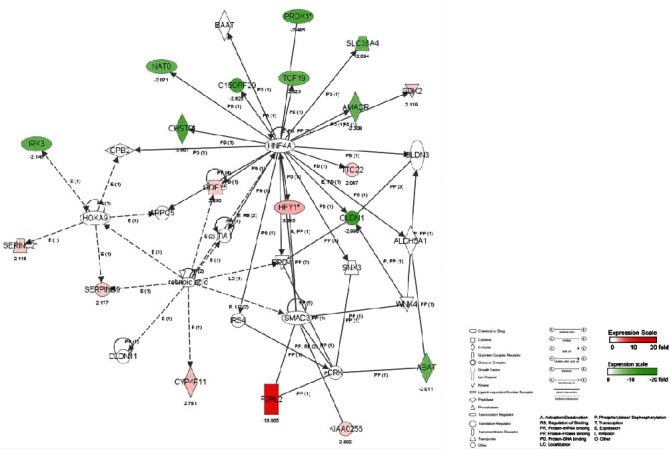
The third most significant molecular network affected by DTS.

There are 35 genes involved in the construction of the network, of which 19 genes are differentially expressed. HNF4A, as the central location gene of this molecular network, controls the transcriptional activity of multiple inflammatory factors and the expression of antioxidant genes. Among them, there are 9 upregulated genes, and the most significant gene with upregulated fold change is F2RL2. There are 10 downregulated genes, and the gene with the most significant downregulation fold change is CLDN1 (Figure 6).

### 3.5. Effect of DTS on Secretion of Inflammatory Cytokines

**Figure 7:**
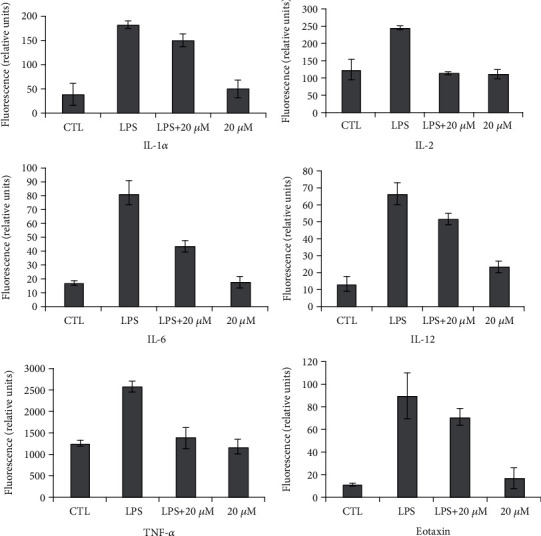
Influence of DTS on the production of inflammatory cytokines.

Since cytokines play an important role in inflammation, the LPS-induced RAW264.7 cell model was constructed to detect the secretion profile of typical cytokines by Bio-Plex 2000 proteome detecting instrument, including the expression levels of 23 inflammatory cytokines, to further verify the results of gene chip analysis and understand the regulation of DTS on the protein expression level of cytokines network. As shown in Figure 7, the most 6 inflammatory factors with significant changes were listed, including IL-1*α*, IL-2, IL-6, IL-12, TNF-*α*, and Eotaxin, which were significantly decreased in a dose-dependent manner after DTS treatment of 10 or 20 *μ* M (P <0.05); meanwhile, the other cytokines had no significant change (data not shown).

IL-1*α* and IL-6 are important proinflammatory factors. LPS was activated NF-*κ*B by activating monocyte MAPK signaling pathway to activate IL-1 gene expression, in which P38 plays an important role in IL-1 secretion. On the other hand, IL-1 also induces the activation of ERK1/2, P38, and JNK, which in turn activates NF-*κ*B and regulates the expression of inflammation-related genes. The effect of IL-6 is closely related to STAT3, which plays an important role in cell proliferation and differentiation. Therefore, IL-6 can not only promote the development of inflammation but also accelerate the proliferation and growth of normal cells and tumor cells. The result indicated that DTS inhibited the expression of pro-inflammatory cytokines in macrophages, which was similar to the result of gene chip. In inflammatory response models, TNF-*α* is an important inflammatory factor, which can bind its receptor TNFR*α* to form trimer-mediated association of cohesive protein TRADD. TRADD recruits the corresponding protein and induces NF-*κ*B activation through TRAF2 and RIP molecules. Therefore, the significantly decreased expression of TNF-*α* in this study indicates that the DTS can inhibit the inflammatory response via NF-*κ*B inactivation.

## 4. Conclusions

Treatment with DADS, DATS, and DTS resulted in the differential expression of genes in liver cancer cells. Most of the genes whose expression changes were greater than 2 times are distributed between upregulated and downregulated expressions 2-3 times. Among the three experimental groups, EGR1, NCF2, and F2RL2 were found while the gene expression changes were more than 4 times. Among the three categories classified according to the GO catalog, the molecular function category contained the largest number of genes with differential expression greater than 2 times, indicating that DADS, DATS, and DTS acted on liver cancer cells and have the greatest impact on their molecular functions. The function with the largest number of genes included is protein binding, indicating that the molecular signaling pathways in cells are significantly affected by DADS, DATS, and DTS. In the DTS treatment group, the gene upregulated and expressed more than 2 times of the phase I coenzyme is NCF2. The genes with upregulated expression of phase II coenzyme or antioxidant proteins more than 2-fold include GCLC, GCLM, SQSTM1, and HMOX1.

Glutamate Metabolism (glutamate metabolism), Acute Phase Response Signaling (acute stress response), NRF2-mediated Oxidative Stress Response (NRF2-mediated oxidative stress response) are the most significant typical signal channels that are changed by DTS. The activation of these signal channel is related to Nrf2-mediated oxidative stress. The ten major molecular functions affected by DTS are Cancer, Cell Death, Cellular Growth and Proliferation, Tissue Development, Cellular Development, Cellular Movement (cell movement), Reproductive System Disease (reproductive system disease), Cell Cycle (cell cycle), Hematological System Development and Function (blood system development and function), and Cell Morphology (cell morphology). The molecular functions with the highest scores affected by DTS are cancer, cell apoptosis, and cell proliferation, which prove that the occurrence or metabolism of these diseases is related to the differential expression of the above-mentioned related genes and the activation of signal channels.

Through the analysis of the molecular network that has the greatest impact on DTS, it is concluded that the molecular functions involved in the first three molecular networks correspond to the signal channels and diseases analyzed above. In particular, the core of the first molecular network is inflammation. The results of Bio-Plex kit for the detection of inflammatory cytokine secretion were consistent with the results of gene chip hybridized for the differential expression of inflammatory cytokine genes, which proved that the results of gene chip were reliable, and also verified that DTS could reduce the secretion of proinflammatory cytokines from the perspective of proteomics.

## Figures and Tables

**Table 1:** Changes of DSs-related gene expression in Hep G2 cells.

	Fold of change	DADS/control	DATS/control	DTS/control
Up	5<	3	3	4
	4	2	6	7
	3	13	18	21
	2	85	99	115
Down	2	25	102	88
	3	0	14	8
	4	0	1	1
	5<	0	1	0
Total	Up	103	126	147
	Down	25	118	97
	Total (±2^3^ fold change)	128	244	244

**Table 2:** Changes in gene expression of drug metabolizing enzymes and transporters in DSs-treated cells.

Gene symbol	Gene title	Fold	Change
Detoxification: phase I drug			
FMO5	Flavin containing monooxygenase 5	3.42	Down
		2.82	
		3.58	
NCF2	Neutrophil cytosolic factor 2 (65 kDa, chronic granulomatous disease, autosomal 2)	4.29	Up
		3.88	
		3.16	
Detoxification: phase II and antioxidant proteins			
SQSTM1	Sequestosome 1	2.32	Up
		2.46	
		2.20	
HMOX1	Heme oxygenase (decycling) 1	2.01	Up
		2.22	
		2.65	
GCLC	Glutamate-cysteine ligase, catalytic subunit	2.32	Up
		2.51	
		2.49	
GCLM	Glutamate-cysteine ligase, modifier subunit	2.36	Up
		2.40	
		2.58	
Detoxification: phase III			
SLC2A2	Solute carrier family 2 (facilitated glucose transporter), member 2	2.17	Down
		3.24	
		3.01	
SLC6A6	Solute carrier family 6 (neurotransmitter transporter, taurine), member 6	3.18	Up
		3.82	
		3.17	

**Table 3:** DSs regulate the expression of inflammation-related genes.

Gene symbol	Gene title	Fold	Change
TLR6	Toll-like receptor 6	2.11	Up
		2.34	
		2.05	
FOS	v-fos FBJ murine osteosarcoma viral oncogene homolog	3.16	Up
		3.88	
		4.13	
SOCS1	Suppressor of cytokine signaling 1	2.07	Down
		2.68	
		2.08	
BCL10	B-cell CLL/lymphoma 10	2.11	Up
		2.32	
		3.05	
TNFAIP3	Tumor necrosis factor, alpha-induced protein 3	2.01	Down
		2.13	
		2.24	
PRKACB	Protein kinase, cAMP-dependent, catalytic, beta	2.17	Up
		2.34	
		2.69	
TNFRSF10D	Tumor necrosis factor receptor superfamily, member 10d, decoy with truncated death domain	2.08	Up
		2.68	
		2.83	

**Table 4:** DSs regulate the expression of genes related to glucose and lipid metabolism.

Gene symbol	Gene title	Fold	Change
GCLC	Glutamate-cysteine ligase, catalytic subunit	2.32	Up
		2.51	
		2.49	
GCLM	Glutamate-cysteine ligase, modifier subunit	2.29	Up
		2.40	
		2.48	
SLC2A2	Solute carrier family 2 (facilitated glucose transporter), member 2	2.17	Down
		3.24	
		3.01	
GCKR	Glucokinase (hexokinase 4) regulator	2.07	Up
		2.04	
		2.95	

**Table 5:** DSs regulate the expression of genes related to cell proliferation and apoptosis.

Gene symbol	Gene title	Fold	Change
F2RL2	Coagulation factor II (thrombin) receptor-like 2	14.14	Up
		15.28	
		15.01	
HSPA1A///HSPA1B	Heat shock 70 kDa protein 1A///heat shock 70 kDa protein 1B	4.29	Up
		4.30	
		3.68	
GCLC	Glutamate-cysteine ligase, catalytic subunit	2.32	Up
		2.51	
		2.49	
GCLM	Glutamate-cysteine ligase, modifier subunit	2.29	Up
		2.40	
		2.48	
SLC2A2	Solute carrier family 2 (facilitated glucose transporter), member 2	2.17	Down
		3.24	
		3.01	
TLR6	Toll-like receptor 6	2.11	Up
		2.34	
		2.05	
FOS	v-fos FBJ murine osteosarcoma viral oncogene homolog	3.16	Up
		3.88	
		4.14	
SOCS1	Suppressor of cytokine signaling 1	2.07	Down
		2.68	
		2.08	
NCF2	Neutrophil cytosolic factor 2 (65 kDa, chronic granulomatous disease, autosomal 2)	4.29	Up
		3.12	
		3.16	
VDR	Vitamin D (1,25-dihydroxyvitamin D3) receptor	2.06	Down
		2.69	
		2.86	
SPP1	Secreted phosphoprotein 1 (osteopontin, bone sialoprotein I, early T-lymphocyte activation 1)	3.20	Up
		2.64	
		2.82	
FOSL1	FOS-like antigen 1	2.19	Up
		2.39	
		2.34	
JAG1	Jagged 1 (Alagille syndrome)	2.76	Up
		3.59	
		4.01	

## Data Availability

All data included in this study are available upon request by contacting the corresponding author.
